# Case of Poisoning by Strychnine, with Experiments on Poisoning by That Substance, and Remarks on Some Parts of the Medical Evidence Given on Palmer’s Trial

**Published:** 1856-10

**Authors:** J. A. Lawrie, J. B. Cowan


*Case of Poisoning by Strychnine, with Experiments on Poisoning by that Substance, and Remarks on some parts of the Medical Evidence given on Palmer's Trial.* By J. A. Lawrie, M. D., and J. B. Cowan, M. D.

(Read before the Medico-Chirurgical Sceiety, 10th June, 1856.)

On the evening of the 11th of June, 1853, a medical gentleman, aged 22, while laboring under considerable excitement, the result of a debauch, called at a druggist's shop in Glasgow, wrote the following recipe, and obtained the mixture:—℞ *Strychniæ pur.* gr. iij., *spirit vin. rect.* ℥ss., *acid sulph. dil.* M. xxx., *aquæ puræ ad* ℥viij., *misce et solve.* He proceeded to the hotel where he resided, retired to his bedroom, undressed, swallowed the whole of the mixture, concealed the empty bottle behind the grate, and went to bed. He slept, according to his own account, but not very soundly, for about an hour and a half, his rest being interrupted by dreams, some of which were of the most pleasing and delightful description. He awoke in a spasm, uttering loud cries which alarmed the household. On the cessation of the spasm he fainted, and, on coming to himself, requested the servant to go immediately for Dr. Montgomery. On that gentleman's arrival, suspecting that a poison had been taken, he immediately dissolved some sulphate of zinc, with which he had provided himself, in water; but on commencing to administer it, the first drop that touched the patient's tongue induced a violent spasm, accompanied with loud shrieks and complete opisthotonos. On the subsidence of this spasm, by introducing his finger to the back of the mouth, and carrying the spout of the drinking cup over it, Dr. Montgomery was enabled to get the emetic partially swallowed. He repeated the dose three or four times in succession. Free vomiting having been induced, the inhalation of chloroform was immediately commenced. At 4 A. M., Dr. Lawrie saw the patient, and, in addition to the continuance of the chloroform, administered a stimulant enema. Between the hours of 4 A. M. and 6.30 A. M., nine spasmodic attacks, more or less severe, occurred. The last of these, which seemed to be induced by the application of a cup to the lips, was very intense and prolonged. The patient started suddenly up in bed, his whole frame being in a state of complete rigidity. The respiration, at first impeded, became suspended, and it was only by the long-continued maintenance of artificial respiration that it was restored. The limbs were rigid, and the fingers clenched. The pupils were dilated. During the spasms, evident relief was afforded by forcible extension of the body. In the intervals there were constant twitches of the extremities. The skin was warm and moist. The pulse was at first extremely rapid, but gradually diminished in frequency. The urine was passed with difficulty. The mind was perfectly collected. From 6.30 A. M. till 2 P. M., the patient was kept by Dr. Cowan almost continuously under the influence of chloroform. The twitches remained till the following day, and the patient then rapidly recovered. It may here be noted that the emetics acted well, that vomiting occurred pretty frequently up till 2 P. M. of the 12th, and that shortly after that hour, when it again happened, some of the undigested dinner of the previous day was found among the rejected matters. It may be stated, that the patient had partaken of a most hearty meal at 4 P. M. of the 11th.

In offering some remarks on the case of Dr. Z., and on the experiments which we have made on animals, we shall take the liberty of comparing them with the symptoms observed during life, and the appearances discovered after death in the case of Cook, as given by some of the medical witnesses on Palmer's trial.

1. As regards the history and symptoms, there is in some respects a remarkable similarity between those of Cook and Dr. Z.

(*a.*) The period which elapsed between the ingestion of the medicine, viz., 1½ hours, corresponds almost exactly. In Dr. Z.'s case, we have only his word to fall back upon; but as he had no motive for deceiving us, we think we are safe in assuming that it is as much to be relied on as any similar statement well can be and quite as much as the case of Cook, or any of those quoted on Palmer's trial. Assuming it, therefore, to be correct, it shows that three grains of strychnine may be swallowed in solution on a full stomach; that the patient, a robust youth, may fall asleep and awake about the end of an hour and a half with the characteristic spasms of strychnine poisoning in their most intense form.

In our experiments on animals, the period from the ingestion to the appearance of the symptoms varied from forty-six minutes, the longest, to seven minutes, the shortest. The following are some of the variations:—

| | | | |
|---|---|---|---|
| Rabbit, ¼ gr. | first symptom in | . . . . . . | 30 minutes. |
| Dog, ¼ gr. | " | . . . . . . | 31 " |
| Dog, ½ gr. | " | . . . . . . | 9 " |
| Dog, ½ gr. | " | . . . . . . | 9 " |
| Dog, ½ gr. | " | . . . . . . | 8 " |
| Horse, 15 gr. | " | . . . . . . | 46 " |
| Dog, ½ gr. | " | . . . . . . | 17 " |
| Dog, ½ gr. | " | . . . . . . | 7 " |
| Dog, ½ gr. | " | . . . . . . | 20 " |
| Dog, ½ gr. | " | . . . . . . | 10 " |

Taking the dogs getting the same dose, we find the average period for the accession of the symptoms to be little more than eleven minutes.

(*b.*) The scream or screams were well marked in both cases. Dr. Z. screamed repeatedly, and so loudly as to terrify the female inmates of the house, so that they would not enter his room. Animals, especially dogs, poisoned by strychnine make no noise. In not one single instance have they barked, howled, or moaned either before or during the paroxysms. In this respect their demeanor contrasts strongly with that which chloroform induces, the latter substance causing a continuous bark or yelp so long as the power of emitting sound continues.

(*c.*) We cannot compare the period of death in the two cases. But if we assume that Dr. Z. would have died but for Dr. Lawrie's interference, we have seven-and-a-half hours elapsing from the ingestion of the poison, and six from the first appearance of tetanic symptoms to the time, if not of probable death, at all events to that at which the symptoms reached their acme.

In animals these periods were:—

| | | | |
|---|---|---|---|
| Rabbit, ¼ gr. | death in | . . . . . . . . | 22 minutes. |
| Dog, ¼ gr. | " | . . . . . . . . | 66 " |
| Dog, ½ gr. | " | . . . . . . . . | 32 " |
| Dog, ½ gr. | " | . . . . . . . . | 18 " |
| Dog, ½ gr. | " | . . . . . . . . | 16 " |
| Horse, 15 grs. | " | . . . . . . | 1 hour 12 " |
| Horse, 57 grs. | " | . . . . . . | 13 hours 36 " |
| Dog, ½ gr. | " | . . . . . . . . | 23 " |
| Dog, ½ gr. | " | . . . . . . . . | 13 " |
| Dog, ½ gr. | " | . . . . . . . . | 49 " |
| Dog, ½ gr. | " | . . . . . . . . | 25 " |

Selecting the dogs getting the same dose, we have an average period of about twenty-five minutes.

(*d.*) The sensitiveness to touch was exceedingly well marked in Dr. Z., especially about the mouth, and had this sensitiveness deterred his medical attendants (especially Dr. Mongomery in the exhibition of the emetic) from devising means to enable him to swallow, and persevering in their employment, the probability is that he would have died. In animals, this acute sensitiveness to touch, or rather to *sudden impressions* of any kind, is well marked. A touch or tap acts like an electric shock. It appears to us, however, that it is the sudden first impression or touch, and not the continuous pressure which causes the shock and the renewal of the spasm, a circumstance which appears to be entirely overlooked by the witnesses on Palmer's trial. In Dr. Z.'s case, Dr. Montgomery was able to give the emetic by passing his finger into his patient's mouth, keeping it there, and pouring the liquid over it. Subsequently, we found that the interposition of a wetted fold of handkerchief pressed in contact with the under lip, enabled him to bear the contact of a cup, and to swallow easily. In dogs, if the finger or rod was continuously pressed against any part of the surface, spasms were not renewed. In connection with this point, much stress was laid by the defence in Palmer's trial, on the fact that Cook asked to have his neck rubbed, and that it was rubbed without causing spasms. Now, we have uniformly found that rubbing any part of the surface in dogs does not cause spasm, and that, so far as we could judge, rubbing or scratching their necks was agreeable to them. As death approaches, the sensitiveness to touch diminishes, and its absence is often the first and best indication that the animal is really dead.

(*e.*) The fact that Dr. Z. vomited, we consider important. Some of the witnesses seem to deny the possibility of vomiting when the system is under the influence of strychnine. Dr. Z. vomited about two-and-a-quarter hours after the ingestion of the poison, and about three-fourths of an hour after the first spasm. This we consider an important fact, and well worthy of being borne in mind, on account of its practical curative importance. Animals, so far as our experiments go, never vomit from strychnine alone; but that they may be made to vomit, is shown by the experiments in which chloroform was given.

The diagnosis of strychnine poisoning is of great importance, and one which deservedly was much insisted on in Palmer's trial. The diseases with which it is most likely to be confounded are tetanus, idiopathic and traumatic, epilepsy, and general convulsions.

1. *Tetanus.*—Will the cause assist us? It will certainly assist, but will not be diagnostic. In idiopathic tetanus it will be of little service, and in the traumatic it ought not to be forgotten, that a lesion so slight as to be overlooked both by the patient and his surgeon (as a blister on the foot from a badly fitting shoe*) will excite the disease, and that we have the best authority for stating that it will supervene upon any state of a wound, from the most recent, as four hours after an amputation, to that in which it is either completely cicatrized, or so nearly healed, as to be quite forgotten by the patient. So far as we can discover, this point was very much overlooked in Palmer's trial, and there is no evidence that the surface of his body was examined to ascertain the existence of a recent abrasion, or newly-closed cicatrix.

On this part of the subject, the important question on Palmer's trial

* Dr, Sym, Ayr, in Glasgow Medical Journal.

was, will a syphilitic sore, primary or secondary, cause traumatic tetanus? It appears to us a remarkable fact, that no recorded case or experience in the affirmative could be adduced; but that is no reason for saying that such an occurrence is improbable, far less impossible.* It would not, in the very least, surprise us to meet with tetanus as the result of an acute chancre or ill-conditioned secondary sore, and we would not fear to hazard our professional reputation on the fact that such cases will yet be met with. We are far from presuming to say that the slight lesions observed, or said to have been observed, on Cook's body, were the cause of his fatal convulsions. We speak of the general etiology of tetanus, and we would remind you that in such cases we must not trust to probabilities. Delegation of tumors would seem, *a priori*, to be a very probable cause of tetanus; experience, however, proves it to be one of the rarest. The same remark applies to parturition.

In forming the diagnosis between strychnine poisoning and tetanus from the symptoms, they may conveniently be divided in those which precede the tetanic paroxysm, those presented during the acme of the paroxysm, and those which follow it. On the first there can hardly be any difficulty. Speaking from our own experience, in every instance the symptoms of tetanus began about the neck and jaw, and are always comparatively, and generally *positively*, slow and progressive; while, after strychnine, the premonitory symptoms are slight, the jaw is unaffected, and the paroxysm occurs early, suddenly, and violently.

The most rapid case of traumatic tetanus which our experience affords, occurred in the Glasgow Royal Infirmary in 1847, in which the symptoms set in three or four hours after amputation of the thigh, and death occurred in eight or nine hours after their accession.

In dogs, the premonitory symptoms are—drawing the tail between the hind legs, uneasiness, unsteadiness, and stiffness of the limbs, altogether giving very much the appearance of an animal frightened, except that he does not growl or bark. He then generally falls on his side, in a sudden, violent, tetanic paroxysm. At times he springs from his feet, and falls flat on his side. At others, he tries to run; his limbs refuse to obey him, are pulled spasmodically under him, and he falls forward on his chest and muzzle.

During the paroxysm, the diagnosis, though more difficult, can hardly be said to be obscure. In strychnine, the paroxysm generally follows a state of comparative relaxation; in tetanus, it is merely the aggravation, often not severe or well marked, of a previously-existing continuous spasm; and almost necessarily, the more intense the continuous spasm, the less marked is the exacerbation. The state of the jaw we consider quite diagnostic. In acute tetanus we have never seen it but so firmly closed, that it could not possibly be wrenched open; while in strychnine, the mouth is very often not closed; and if closed, however spasmodically, can always be easily and widely opened. In proof of this, we would adduce the second experiment on the horse, in which, an hour or two before he died, while the paroxysms were on him, and after he had

* Since the above was written, a case has been published in the Medical Times and Gazette of 7th June.

swallowed 47 grains, we found no difficulty in giving 10 more in solution, through a common beer bottle. This in tetanus would be impossible.

The account which we have given of the state of the jaw seems to have been overlooked by the great majority of the medical witnesses. Indeed, Dr. Todd is almost the only one with whose evidence our experience agrees. Dr. Corbett says, that in the case of French, "there was not what is called locked jaw in ordinary tetanus."

The *risus sardonicus*, so much insisted on by Mr. Solly, we cannot look upon as of any real importance, more especially in such a case as Cook's at midnight in November, with one candle in the room, and the face turned from the surgeon.

The effect of sudden impressions, in aggravating or inducing the strychnine paroxysm, is very marked, and has already been adverted to.

In the intervals of the paroxysm, the diagnosis is easy. In tetanus there are slight remissions, but we have never seen intermissions; and, as a general rule, the more rapid and acute the attack, the less marked are the remissions. The mouth can never, in our experience, be opened. In strychnine the intermission is complete. The mouth opens; in dogs, the tongue hangs out, and the breathing is rapid and panting; at times excessively so. In tetanus the tongue is almost always lacerated by the teeth. In strychnine, however sudden and violent the paroxysm, we have never seen it injured.

In epilepsy, tetanic rigidity, properly so called, can hardly be said to occur. The rolling of the head, beating with the arms and hands, foaming at the mouth, and usually complete unconsciousness, not only during, but after the paroxysm, must be quite diagnostic. The state of the mind during the paroxysm must not be too much insisted on, because we believe that the patient is not conscious during the violent strychnine paroxysm (Dr. Z. certainly was unconscious,) and because cases of epilepsy are said to be recorded, in which consciousness was not lost. A careful search, however, has not enabled us to discover any account of these.

General convulsions are easily distinguishable from the spasms of strychnine, by the rapidity of their accession and of their cessation, by the irregularity of the alternate contractions and relaxations of the voluntary muscles, and by the absence of twitches in the interval of the paroxysms.

*Post-mortem Rigidity.*—We need hardly remind this society that the general law is, that the muscular system becomes lax immediately after death, whatever the cause of death may have been, and remains so for a period, varying with the cause of death, the age and previous strength, the season of the year, and many other circumstances. As the body cools, the rigor mortis sets in, and varies in the rapidity of its accession, its intensity and duration, with circumstances and conditions analogous to those above stated. In our experiments the above law has invariably held true. In many of our cases the animal died quite flaccid, and so quietly, that it was not easy to mark the moment at which death occurred; indeed, in one experiment, the body was, by mistake, opened before life was extinct. In others, death took place during a tetanic paroxysm, and suddenly, but in every instance death was followed by complete flaccidity. As a general rule, the rigor mortis set in early (say in 30 minutes,) but we doubt very much if this is earlier than when death ensues from violence or other unnatural causes. The rigor mortis did not appear to us to be unusually great, or of unusual persistence. Altogether we have not observed any circumstances connected with the state of the muscular system immediately before, at, or after death, on which reliance could be placed as indicating, much less as certainly diagnostic of, the cause of death.

With these convictions we cannot refrain from expressing our surprise at the discrepancy of the medical evidence on this point, and at the great importance which the bench, especially, seemed to attach to it. Nothing can be more loose and uncertain than the opinions of nurses, body dressers, and undertakers, on the comparative amount of rigidity which they allege they have observed, more especially when they speak from memory at the distance of months, and when they have been made aware that their evidence is considered important. It is fearful to think that the innocence or guilt, life or ignominious death of a fellow-man, can in any way be made to depend on such vague uncertainty. But even supposing that all the circumstances connected with the muscular rigidity had been accurately ascertained and correctly sworn to, it appears to us that by far too much importance was attached to it. We doubt very much if, at this moment, any reliable evidence, either recorded or oral, could be adduced in Great Britain, on the difference as to muscular post-mortem rigidity between spasmodic and non-spasmodic disease, much less between any two diseases of the same order. We have seen a good deal of tetanus, and thought we had observed it pretty closely, but if examined as to our personal observation on these points, we should at once confess our ignorance. The truth is, that any observations which are not absolutely accurate are worse than useless, and that the number of medical men who have made this subject one of accurate personal observation is very small.

We were surprised that no allusion was made to the great rigidity which follows cholera; or to the fact, that, in many instances of death from ordinary causes, it is easier to rupture tendons than to bend articulations. Any one who has operated frequently for stone on the dead subject, must be aware that peculiarly contracted states of the hands and fingers is very common, and that the strength of two muscular men is often requisite to bend the knee or hip joints.

That dead bodies are found stiff in the exact position in which death occurred, can be accounted for in two ways:—

1. In the majority of deaths, muscular power has so completely failed before life was extinct, that the laws of gravitation produce no change on the position of the bodies, and, if undisturbed, they stiffen in the precise position in which they died. That this is the general rule, we have no doubt.

2. There is reason to believe that in some rare instances, when death occurs during convulsive spasm, the spasm of life is prolonged into the rigor mortis without any intermediate relaxation. If we mistake not, one of the most authentic and remarkable examples of this kind occurred in this city some years ago, in the practice of the late Dr. M'Gregor, in which the death-grasp of the dying man refused to relax its hold after death, until the tendons of the fingers were severed by the knife. The death occurred from a sudden attack of epilepsy.

*Post-mortem Appearances.*—The heart. When examined immediately after death, the right side of heart was generally found contracting very markedly, the left side very slightly. The cavities were invariably full, those of the right side being distended. In animals examined after a lapse of time, the cavities of the right side of heart were always found full of, either completely or partially coagulated blood, the walls of right ventricle being soft, while the left ventricle was less full, and its walls firm and contracted. In one case the left auricle was perfectly empty, but it generally contained a small quantity of blood. In the horse poisoned by 57 grains, the examination was made eleven hours after death. The right side of heart was turgid, both cavities being filled with dark blood, partly coagulated and partly fluid, and traceable into large vessels. The left side was not distended, but the ventricle contained a large fibrinous clot continuing into auricle. In a case, to be afterwards adverted to, in which a grain of strychnia was given at 8.55 A.M., and the administration of chloroform continued till nearly 4 P.M., without the occurrence of a spasm; on its withdrawal, the tetanic spasms manifested themselves, and the dog died. The heart was found enormously distended, and the pericardium contained a large quantity of serous fluid.

The empty state of the heart in Cook's case was considered a very favorable point for Palmer by his counsel and agents. The Crown, however, succeeded in proving that, in two out of the three cases sworn to in court, those of Agnes French and of Mrs. Smith, the viscus was empty. In the third, the Leed's case, the precognition on this point had not been satisfactory, for the Crown counsel, in his examination of Mr. Morley, skilfully avoided all allusion to it. The prisoner's counsel, in cross-examination, elicits the admission that, "the head being opened first, a good deal of blood flowed from the heart, and that, consequently, he was uncertain as to whether the heart was full or empty;" while Mr. Nunneley, a witness for the defence, and who had assisted Mr. Morley at the examination, says—"We had no doubt of the heart being full, but the blood was drained away, owing to the opening of the head, and by the cutting of the large vessels."

With regard to animals, all the medical witnesses examined as to the results of experiments, with a single exception, admit that the heart is always full, especially the right side; those for the prosecution couching the terms of their answer in a milder, those for the defence in a more decided form. The exception is Dr. Todd, who says, "the right side of heart was *not* generally full."

The lungs were found in the horse deeply congested throughout, and the pulmonary vessels contained clots. In four dogs they were also found more or less congested, while in the remainder they were normal in appearance.

The stomach of the horse was of the usual size. There were a few very prominent rugæ near pylorus, and, as is not an uncommon occurrence, portions of the lining membrane were digested, and a few worms clung to the walls. The stomachs of all the dogs examined were contracted when the viscus was empty, or nearly so; but in two cases, in which it was distended with food, there was no diminution of size. The contraction was very marked near the pyloric orifice, and in this situation, in several instances, there were marks of congestion, and in all peculiar earthworm-like rugæ. These rugæ were more fully developed by the operation of washing. We have since examined the stomachs of dogs not killed by strychnine, and found similar appearances, though not so well marked as in these specimens.

The liver was healthy in the horse, congested in one dog only. In the horse the large intestines were of normal size, while the small bowels were said to be more contracted and firmer than usual. In two of the dogs the latter were decidedly contracted.

The brain and spinal marrow throughout its entire extent were carefully examined in four cases, but did not present the slightest deviation from their ordinary appearances.

After sacrificing what some may possibly consider a more than sufficient amount of animal life, in ascertaining the symptoms during life and appearances after death, we turned our attention to the more pleasing task of discovering an antidote. The beneficial results which followed, or seemed to follow, the inhalation of chloroform in Dr. Z.'s case, naturally led us to try it in the first instance.

*Experiment* 1.—To a small-sized terrier, ½ gr. of strychnine was given in pill. In eight minutes the tetanic rigidity was complete. Chloroform was given by inhalation, and artificial respiration attempted by pressure on the ribs. In nine minutes the animal died. Results almost precisely similar followed in the second and third experiments of this series.

These experiments showed, that if we waited till the tetanic effect of a poisonous dose was fully established, the respiration was so weak that chloroform could not act. To obviate this, the fourth and fifth experiments were tried.

*Experiment* 4.—At 8.30, ¼ of a grain in pill was given to a small dog, and the administration of chloroform immediately commenced. In about three minutes it was under its influence, at first struggling considerably, and then barking and howling continuously. A slow regular paddling motion of the limbs then commenced, gradually becoming more rapid. On the animal emerging from the influence of the vapor, this ceased, but immediately began on its re-administration. At 9.36 vomiting occurred. It was left living at 10.15, having exhibited no symptoms of spasm, and shortly after 12 was found dead, the body being slightly rigid.

*Experiment* 5.—¾ of a grain in pill were given to a dog at 9, when under chloroform. After twenty minutes, the same paddling motion observed in last experiment began. Chloroform continued till 10.15, no spasm occurring. Found dead at 12, and body quite warm and flaccid.

These experiments having satisfied us of the power of chloroform to retard the action of strychnine, we resolved to prolong its continuous influence almost indefinitely, and with this view instituted the following:—

*Experiment* 6.—At four minutes to 8 A.M., about half a grain of strychnine in a crumb of bread was given to a young full-sized mongrel terrier, and chloroform immediately administered. It was easily brought under its influence, and then began to bark, and latterly to moan. At a quarter to 10, appearing stupified, very small quantities of the vapor were given. At 11.10 passed urine; at 11.40 recommenced to bark; at 12 passed urine; at 12.50 had an alvine evacuation. Again made water at 1.15; vomited at 3; lay quietly breathing till 3.40. The gentleman who had kindly undertaken to administer the chloroform now thought the danger was over, and therefore discontinued the use of the chloroform, and gave it some bread, which it ate eagerly. At 3.55 had a violent spasm. The spasms continued in rapid succession til 4.30, when the animal died, the body being quite flaccid.

*Experiment* 7.—One grain in crumb of bread given to a large pointer bitch at 8.55, and chloroform immediately administered. When under its influence, she moaned loudly. The pupils were intensely dilated. Vomiting occurred at 9.45. The animal continued perfectly quiet till 3.45, when she ate some bread and walked about. Eight minutes subsequently she was seized with spasm, and died at 3.55. The body was opened shortly after death, and the heart found enormously distended.

Our mortification at the untoward results of these experiments was very great. As the point to be ascertained was of great interest and importance, the following experiment was begun this morning:—

*Experiment* 8.—At 8.19 half a grain was given pure to a black terrier, and chloroform inhalation immediately begun. At 9.50 it had a spasm of no great violence, and died.

*Experiment* 9.—At 8.20 half a grain of strychnine in solution was given to a house terrier. At 4.15 P.M., it was alive, breathing quietly. It lived till 11.35 P.M., being fifteen hours and fifteen minutes.

The result of this experiment, so far as it went, was very satisfactory, proving that chloroform can arrest the influence of the poison. Sulphuric ether was not found on trial to be at all equal to chloroform.

*Glasgow Medical Journal.*

